# Prediction and Structural Comparison of Deleterious Coding Nonsynonymous Single Nucleotide Polymorphisms (nsSNPs) in Human LEP Gene Associated with Obesity

**DOI:** 10.1155/2019/1832084

**Published:** 2019-12-04

**Authors:** Hind Bouafi, Sara Bencheikh, AL Mehdi Krami, Imane Morjane, Hicham Charoute, Hassan Rouba, Rachid Saile, Fouad Benhnini, Abdelhamid Barakat

**Affiliations:** ^1^Laboratoire de Génomique et Génétique Humaine, Institut Pasteur du Maroc, Casablanca, Morocco; ^2^Laboratoire Biologie et Santé, Centre de Recherche Santé et Biotechnologie, Faculté des Sciences Ben M'Sik, Hassan II University of Casablanca, Morocco; ^3^Laboratoire de Signalisation cellulaire, Faculté des Sciences Meknès, Université Moulay Ismail, Morocco

## Abstract

Leptin is a peptide hormone that regulates fat stores in the body and appetite by controlling the feeling of satiety. This hormone is secreted by the white adipose tissue and plays a role in the storage and mobilization of fatty acids. Mutations of the LEP gene have been associated with obesity in different populations; it is a multifactorial disease that constitutes a major public health problem. In this study, we evaluated the impact of missense SNPs in the LEP gene extracted from dbSNP using 8 computational prediction tools. Out of the total of 4337 SNPs, 93 were nsSNPs (nonsynonymous single nucleotide polymorphisms). Among 93 nsSNPs, 12 (S46L, G59S, D61N, D100N, N103K, C117S, D76V, S88C, P90R, I95N, L161R, and R105W) variants were predicted to be the most deleterious by prediction software. On these 12 deleterious SNPs, 8 variants (S46L, G59S, D61N, D100N, N103K, C117S, L161R, and R105W) were located in the conserved positions and showed a decrease in structure stability which was evaluated by I-Mutant and Mupro. Then, by analyzing the different interactions between different amino acids in wild and mutated proteins, we assessed the structural impact of the deleterious modifications using the YASARA software. Among 8 deleterious nsSNPs, we revealed structure changes in the 6 variants S46L, G59S, D100N, L103K, R105W, L161R, two of which R105W, N103K were previously reported as associated with obesity. Our study suggests 6 deleterious mutations could play an important role in contributing to human obesity and worth to be included in association and functional studies, then may be a drug target.

## 1. Introduction

Metabolic syndrome (MetS) is a worldwide epidemic complex disorder determined by a cluster of interconnected factors, which are dyslipidemia, hypertension, and dysregulated glucose homeostasis, while abdominal obesity and/or insulin resistance (IR) are the main manifestations of the syndrome. All of these factors increase the risk of cardiovascular atherosclerotic diseases and type 2 diabetes mellitus [1]. Most of the epidemiological studies show that MetS's prevalence ranges between 20% and 45% of the population [2].

Abdominal obesity has indeed recently gained increasing and special attention as the most prevalent manifestation of metabolic syndrome according to the National Cholesterol Education Program Adult Treatment Panel III (NCEP-ATPIII) and the International Diabetes Federation (IDF) [3]. Moreover, in 2016, the number of overweight people reached as high as 1.9 billion adults worldwide of whom more than 650 million were obese [4].

The imbalance between caloric consumption and energy expenditure mainly characterizes obesity, which is defined, according to the World Health Organization by the calculation of the body mass index (BMI) determined by the following formula: weight in kilograms/height in meters 2, as well as by measuring waist circumference. Indeed, a BMI of 30 or more and a waist circumference greater than 80 cm in women and 94 cm in men indicate obesity [5]; furthermore, it becomes morbid when the BMI value exceeds 40 [4].

This global epidemic disease represents the fifth leading cause of death in the world. This is explained by the fact that it is associated with various dangerous diseases, such as cardiovascular disease (CVD), hypertension (HT), type 2 diabetes mellitus (T2DM), hyperlipidemia, stroke, certain types of cancer, sleep apnea, liver, and gall bladder disease [6].

In addition obesity is a dysfunctional marker of fat tissue caused by interactions between environmental (inadequate diet, sedentary behavior, psychological, and sociocultural beliefs) and genetic factors [7]. While the hereditary nature of the corpulence is indisputable, it has been reported, with expansion of genome wide association studies, that more than 50 genes are directly related to obesity. Among these genes, the LEP gene is considered as one of the major genetic factors involved in the obesity pathogenesis [3, 7–10].

The LEP gene, also known as Ob gene, is located on chromosome 7q31.3; it contains 3 exons separated by 2 introns [11] and codes for a mature and functional protein, the Leptin protein. This protein belongs to the family of long-chain helical cytokines and contains 146 amino acids. The Leptin protein is a metabolic and neuroendocrine hormone that is mainly secreted in the white fat tissue by the differentiated adipocytes. It is also an essential factor for the brain development and the formation of hypothalamic pathways, thus playing a key role in the regulation of body weight by inhibiting food intake and stimulating energy expenditure [12].

The Leptin communicates the state of the body energy to the central nervous system. This action is predominantly done via the long form Leptin receptor b (LepRb) expressed mainly in the hypothalamus neurons, considered as the principal target for central Leptin action [13]. The LepRb is responsible for the main effects of Leptin on the energy homeostasis, through its Leptin binding domain and its extended intracellular signaling domain. Mechanisms of Leptin Action and Leptin homeostasis control of feeding are carried out by its anorectic action. The Leptin protein regulates the orexigenic NPY/AgRP neurons by their inhibition and the anorexigenic POMC/CART neurons by their activation, furthermore it regulates the expression and the release of their neuropeptides and, therefore it leads to the food intake decrease and the energy expenditure increase [14]. Moreover, the binding of Leptin to its LepRb receptor triggers the activation of a signaling pathway by the activation and the phosphorylation of JAK2 tyrosine kinase associated to the LepRb receptor, which itself activates the phosphorylation of three tyrosine residues on LepRb, of which Y1138 activates STAT3 signaling and mediates the main effects of Leptin on the energy homeostasis [15].

In addition, it has been shown that clusters of LepRb located in extrahypothalamic sites are crucial as mediators of the anorexic action of the Leptin and thus the body weight control. Attached to these sites, Leptin controls different aspects of the energy homeostasis, including anorexia circuits, hedonic feeding, energy expenditure, and glucose homeostasis [16].

It is now confirmed that the dysregulation of central neural circuits is one of the main causes of obesity, which may be the result of the lack or the absence of Leptin signaling caused by LEP gene mutations [16, 17].

However, it has been shown that in obese people, there is a high level of Leptin which correlates positively with adipose tissue and does not have any effect of reducing nutrition and thus preventing obesity. This has been explained by a physiological mechanism of resistance to the catabolic effects of the Leptin action on obesity. This may be due to an alteration of the Leptin transportation, a cellular LepRb signaling perturbation and rarely a genetic alteration of LepRb [19].

Bioinformatics hold a central place in modern biology, mainly involving computer sciences, mathematics, and statistics in order to analyze and treat biology information. Bioinformatics dispose of many tools vaguely implicated in the analysis of protein structure and function as well as the identification of multiple sequence alignment of homologous proteins, the search for sequence patterns and the evolutionary analysis.

Biocomputing plays a key role in understanding genomic variations implication, especially single-nucleotide polymorphisms (SNPs), which represent the most frequent genetic variations in the human genome.

The nonsynonymous SNPs (nsSNPs) are the single nucleotide variations that affect the coding region of the protein and modify the mutated site-encoded amino acid, which may lead to a structural modification of the mutated protein, and may thus cause function alteration. These variations can be the origin of complex and frequent diseases such as obesity, diabetes, and hypertension [20].

Hence, the aim of our work was to perform a computational analysis in order to determine the most functionally deleterious SNPs of human LEP gene using a cluster of mutation prediction tools, conservation and stability in addition to a structural analysis.

## 2. Materials and Methods

**Figure 1:**
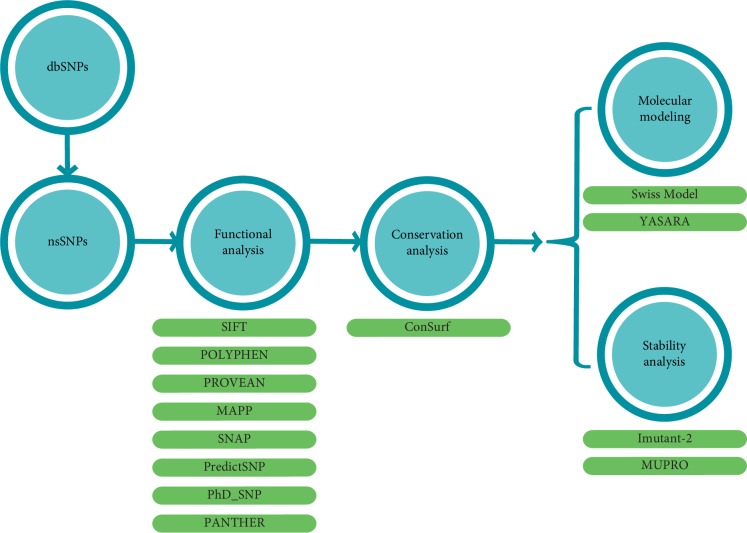
Computational methodology used for the functional nsSNPs analysis.

Our computational strategy was similar to that used by different previous studies [21–23] which includes the prediction of deleterious nsSNPs in LEP gene from the public datasets using various bioinformatics tools. High-risk nsSNPs were further selected for conservational, stability, and structural analysis. The workflow in Figure 1 illustrates the overall computational process of identification and characterization of candidate functional nsSNPs in LEP gene (Figure 1).

### 2.1. Dataset Collection

Human LEP gene information data were retrieved from OMIM database (Online Mendelian Inheritance in Man) (https://www.omim.org/), while the SNPs information of the LEP gene was retrieved from the NCBI dbSNP database (https://www.ncbi.nlm.nih.gov/snp/).

### 2.2. Prediction of Deleterious nsSNPs

SIFT (Sorting Intolerant From Tolerant) was used to differentiate between tolerant and intolerant coding mutations. This software is based on multiple alignment information to predict tolerated or deleterious substitution for every position of the query sequence; this server calculates the probability of an amino acid to be tolerated at a specific position. Substitutions with probabilities less than a tolerance index of 0.05 are predicted to be intolerant or deleterious; those superior or equal to 0.05 are predicted to be tolerated [24]. PolyPhen2 (Polymorphism Phenotyping) is a software which predicts the possible effects of an amino acid substitution on the structure and the function of a protein by using structural and comparative evolutionary considerations. This prediction is based on several features including the sequence, phylogenetic and structural information that characterize the substitution. Each amino acid substitution has a qualitative prediction (“probably damaging”, “potentially damaging”, “benign” or “unknown”). The PolyPhen-2 score varies from 0.0 (tolerated) to 1.0 (deleterious) [25].

Other software were used to confirm the impact of deleterious mutations on the structure and function of Leptin: MAPP (Multivariate Analysis of Protein Polymorphism) [26] predicts the functional effect of amino acid substitutions based on the quantification of physicochemical deviation present in a column of a protein sequence alignment. The greater is the calculated deviation, the higher is the probability that amino acid substitution affects the protein normal function. PANTHER (Protein Analysis Through Evolutionary Relationships) [27], uses evolutionary conservation of amino acid to predict pathogenic coding variants. It uses an alignment of evolutionarily related proteins to estimate how long the current state of a given amino acid has been preserved in its ancestors. The longer the preservation time, the higher the probability of functional consequences. PhD-SNP (Predictor of human Deleterious Single Nucleotide Polymorphisms) [28], is a trained classifier that can predict whether a nsSNP is disease related or not based on a machine learning technique. Starting from the protein sequence, this Support Vector Machine (SVM) classifier can distinguish between deleterious and neutral nsSNPs. SNAP (screening for nonacceptable polymorphisms) [29], a neural-network based classifier for the prediction of the functional effects of single amino acid substitutions. SNAP utilizes sequence information, as well as functional and structural annotations to predict whether a nsSNP is likely to disrupt protein function. PredictSNP [30], is a consensus classifier that combines the output of six different prediction tools (MAPP, nsSNP Analyzer, PANTHER, PhD-SNP, PolyPhen-1, PolyPhen-2, SIFT, and SNAP) to evaluate the effect of nsSNPs on protein function. PROVEAN [31], is a sequence based prediction tool that predicts the functional effect of protein variations. This tool employs an alignment-based score that measures the change in sequence similarity of a protein before and after the introduction of an amino acid variation.

### 2.3. Sequence Conservation

ConSurf is a web server used to analyze amino acids conservation (https://consurf.tau.ac.il). This algorithm identifies the functional regions of a protein by evaluating the degree of this conservation. The grades varying from 1 to 9 indicate the extent of conservation of the amino acid throughout evolution. Consequently, grade 9 refers to the highest conserved residue, while grade 1 represents the least conserved residue [32].

### 2.4. Prediction of Change in Protein Stability

I-Mutant (https://folding.biofold.org/cgi-bin/i-mutant2.0) is a neural network used for automatic prediction of SNPs that affect the protein stability. It also measures the degree of protein destabilization [33] and gives the predicted free energy change value (DDG) and the sign of the prediction: Increase or Decrease. The DDG value is the Gibbs free energy value from the mutated protein minus the Gibbs free energy value from the wild type to Kcal/mol. A DDG value below 0 means that the stability of the protein has decreased, whereas a DDG superior to 0 means it has increased [34].

The stability was also evaluated by Mupro tool (https://mupro.proteomics.ics.uci.edu). This web server is developed by two machine learning methods: Support Vector Machines and Neural Networks that predict the incidence of single site amino acid mutation on protein stability. Both of them were trained from a large set of mutations data with an accuracy superior to 84% and calculated a score that ranged from −1 to 1 as the prediction reliability [35].

### 2.5. Analysis of Structural Impacts of SNPs

The FASTA sequence of Leptin protein was extracted from Uniprot Protein Database (https://www.uniprot.org/), while the 3D structure was built using Swiss Model web server (https://swissmodel.expasy.org) [36]. This server generates reliable structure models by giving their QMEAN and the degree of identity with the experimental protein sequence. The model with a QMEAN of −1, 82 and a sequence identity of .99, 32% was selected. The PDB code of this model is 1AX8. Mutant models were constructed manually from the native protein FASTA file. Then, The wild type and the mutant structures were subjected to the energy minimization by the Gromacs 5.1.4 program [37]. At the end, structural analysis was carried out using YASARA software allowing molecular visualization and comparison of 3D structure between the wild type and the mutant proteins [38].

## 3. Results

### 3.1. SNP Datasets

**Figure 2:**
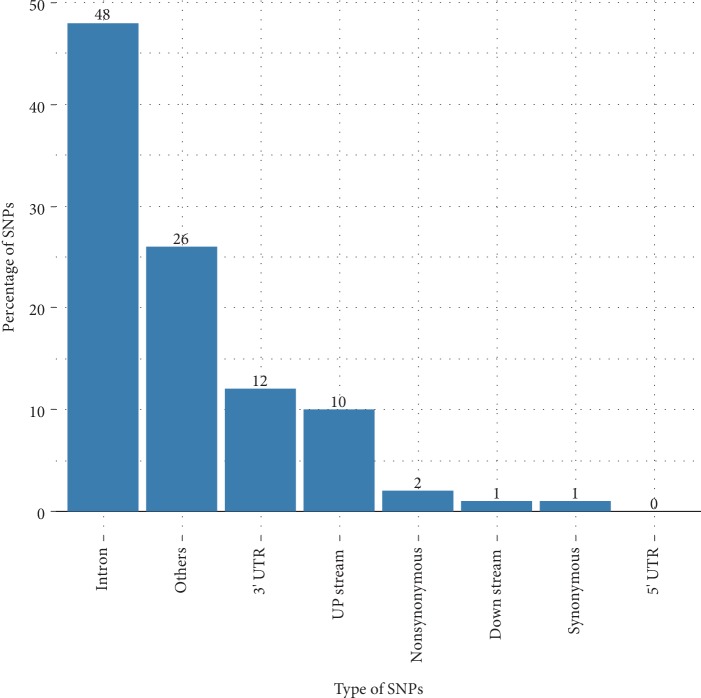
Distribution of SNPs present in the LEP gene.

We collected a total of 4337 SNPs, including 93 nsSNPs, 72 were synonymous variants, and 733 were located in 3′ UTR region and 14 in 5′ UTR region. In addition, 2930 were in the intronic region, 595 and 91 were, respectively, at the upstream and downstream regions of the gene. Others section include diverse variants such as inframe deletion, coding sequence variant, and splice acceptor variant. Results are shown in Figure 2.

### 3.2. Prediction of Deleterious NsSNPs

We were interested in the nsSNPs by predicting their structural and functional effect on the Leptin protein. Among the 93 nsSNPs, only 9 nsSNPs (S46L, G59S, D61N, D100N, N103K, C117S, D76V, L161R and R105W) were selected as being probably or totally deleterious by SIFT and PolyPhen (Table 1). The values filtered by SIFT were between 0 and 0.02, while those filtered by PolyPhen were between 0, 60, and 1. However, the nsSNPs S88C, P90R, and I95N were predicted to be less deleterious by PolyPhen, but other prediction software confirmed their deleterious effects (Table 2). In total, 12 nsSNPs (S46L, G59S, D61N, D100N, N103K, C117S, D76V, S88C, P90R, I95N, L161R, and R105W) were selected to be the most deleterious by prediction software.

### 3.3. Analysis of Conservation

**Figure 3:**
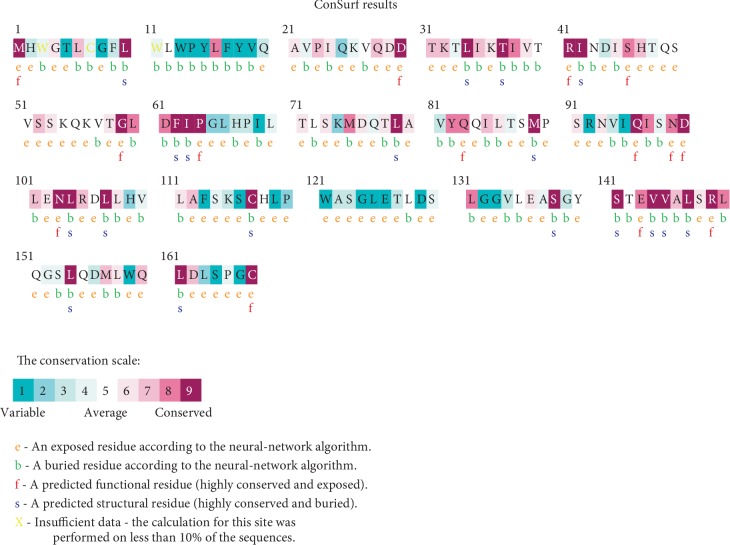
Evolutionary conservation of amino acids in the Leptin protein determined by ConSurf server.

The results of the Consurf analysis showed that within the 12 nsSNPs, only 8 deleterious missense SNPs were located in conserved regions with a score of 7-8-9. Among these, 4 were predicted as functional residues and 2 were predicted as structural residues. The 4 residues D76V, S88C, P90R, and I95N were predicted as variable residues; therefore, there were not selected for the further analysis. Results are shown in Figure 3.

### 3.4. The Impact of Predicted Deleterious Mutations on Leptin Protein Stability

The evaluation of the effect of the 8 nsSNPs predicted as deleterious from the previous steps on the stability of the Leptin structure was done by I-Mutant and Mupro. All of these showed a decrease in structure stability and were selected for the structural analysis. The results are shown in Table 3.

### 3.5. Structural Analysis

The structure of the Leptin protein was analyzed by YASARA software, which identifies the different types of bonds between the different residues generating the 3D structure of the protein. These bonds play a major role in the stability and the folding of protein while each disruption in these interactions affects structure and might interrupt the function of the Leptin.

The different structural interactions that exist in the wild-type protein were compared with the mutated proteins. The result showed that there is a variation in the number of different types of interactions between the native and 6 Leptin mutants.

**Figure 4:**
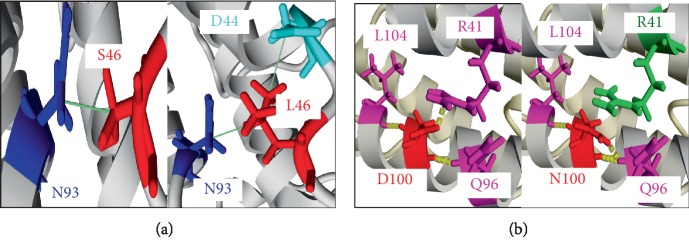
Comparison of the native leptin protein structure and two mutant forms. (a) S46 (wild type-Leptin) and 46 L (variant protein). (b) D100 (wild type-Leptin) 100 N (variant protein). Residues substituted are showed in red, residues involved in hydrogen bonds are marked in magenta, residues participate in hydrophobic interactions are indicated in blue, the residues which lost a hydrogen bonds and/or hydrophobic interactions are marked in green, the new residues appeared are indicated in cyan. Hydrogen bonding are marked by yellow dashed lines and hydrophobic interactions are showed by green lines.

For the S46L variant, a single hydrophobic binding, was identified in the wild-type protein between Serine at position 46 and Asparagine at position 93, while a new hydrophobic binding between leucine at position 46 and Aspartate at position 44 was added to the mutated protein whose Serine was replaced by Leucine, in addition to the hydrophobic binding cited in the wild-type protein (Figure 4(a)).

Regarding the G59S variant, Guanine had a single hydrogen bond with the Leucine residue in the wild-type protein, which disappeared in the mutated protein (Supplementary Material, [Supplementary-material supplementary-material-1]).

Regarding the D100N variant, the wild-type protein had 3 hydrogen bonds formed with the 3 Leucine residues, Glutamine and Arginine, respectively, at positions 104, 96, and 41. The mutated protein kept the same residue at position 104 with the addition of another hydrogen bond in position 96 plus the disappearance of the hydrogen bond in position 41 (Figure 4(b)).

For the N103K variant, the wild protein showed the presence of 2 hydrogen bonds; one with the Leucine residue in position 107 and the other with the Asparagine residue in position 99. In addition to these two bonds in the mutated protein, there was an appearance of a hydrogen bond with the Aspartate residue at position 100 as well as a hydrophobic bond with the Leucine residue at position 34 (Supplementary Material, [Supplementary-material supplementary-material-1]).

At position 105, the Arginine residue had 4 hydrogen bonds with the Leucine, Histidine, Glutamine, and Aspartate at positions 101, 109, 83, and 76, respectively, and a single hydrophobic binding with Leucine at position 79. In the mutated protein in which Arginine was replaced by Tryptophan, bonds at positions 79, 76, and 83 were lost, while no changes were observed in positions 109 and 101 (Supplementary Material, [Supplementary-material supplementary-material-1]).

The L161R variant, the wild protein had also 2 hydrogen bonds, one with the Serine residue at position 164 and the other with the Methionine residue at position 157, in addition to 2 other hydrophobic bonds; one with the Threonine residue at position 31 and the other with the Isoleucine residue at position 35. However, all these bonds disappeared in the mutated protein (Supplementary Material, [Supplementary-material supplementary-material-1]).

In contrast, the variants D61N and C117S did not reveal any changes between the mutated and the wild-type protein.

## 4. Discussion

Many genomic variants especially SNPs have been identified through high-throughput technologies. The National Biotechnology Information Center (NCBI) has created the dbSNP database that contains nearly 44 million validated human SNPs [39]. One of the interests of bioinformatics is to differentiate between the deleterious and neutral SNPs [40]. Particularly, *In silico* analysis of nsSNPs provides an essential tool to select potential variants that can be associated with a disease.

Since its discovery in 1994, the Leptin protein has attracted much attention as an essential central and peripheral signal to maintain the energy homeostasis. Moreover, many studies have demonstrated the association of the LEP gene mutations and obesity in obese patients [3, 41–43]. This study ran a computational analysis of nsSNPs of the LEP gene.

The nsSNPs of the LEP gene were collected from the dbSNP database. These variants were analyzed by the different prediction algorithms in order to measure their probabilities for being deleterious by modifying the amino acids. Evolutionary information represents widely the most useful feature for such a prediction task [40]. With the aim of strengthening the nsSNPs' prediction and bias avoiding, several programs were solicited [44].

From 93 nsSNPs, 8 variants (S46L, G59S, D61N, D100N, N103K, R105W, C117S, and L161R) were predicted to be the most deleterious and located in conserved positions. According to the study established by Grasso et al., the activity of Leptin is localized in parts of the areas between residues 106–140 [45], which includes the variant C117S of our collection. While disulfide bonds and their interactions are strongly conserved in nature, their change is expected to alter the protein folding and thus its function. Therefore, the variant C117S is suspected to be a potential candidate for Leptin destabilization.

**Figure 5:**
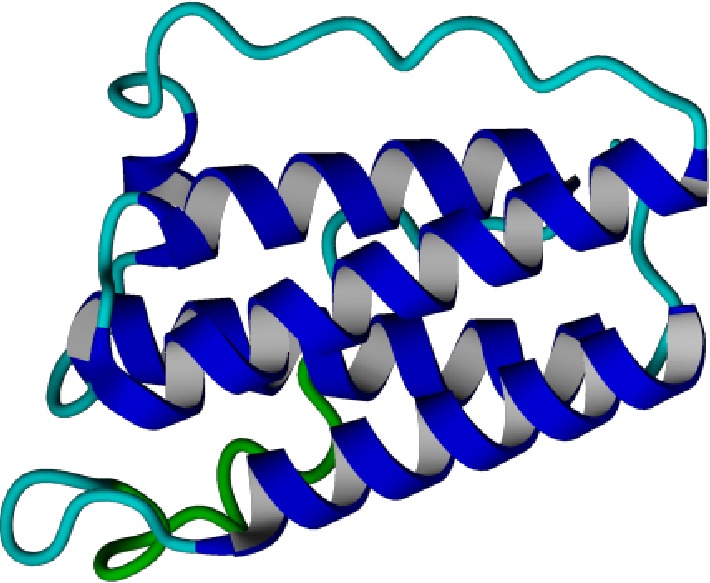
*In silico* modeled structure of LEPTIN protein. The three-dimensional model of LEPTIN generated using Swiss Model Software.

The Leptin protein consists of four antiparallel *α*-helices connected by two long crossover links and one short loop, arranged into a left-hand twisted helical bundle (Figure 5) [46]. The refolding is maintained by different interactions ensuring their stability. After the first step, I-mutant and Mupro were used to evaluate the protein structure stability. These tools predicted that the 8 nsSNPs identified as deleterious decrease the stability which can significantly affect the protein structure. According to Wang and Moult study, approximately 80% of their disease associated nsSNPs dataset generated protein destabilization [47]. Furthermore, Yue and Moult estimated that up to 25% of nsSNPs might alter the protein function in the human population by affecting the protein stability [48]. In this case, the deleterious nsSNPs may lead to the dysfunction of the mutant Leptin protein, which can affect energy homeostasis. Indeed, mutations in the LEP gene were reported to decrease the Leptin protein secretion or cause its inactivation leading to the signaling pathway damage, which can be manifested by physiopathological abnormalities such as homeostatic feeding, energy expenditure, and glucose homeostasis dysregulations [16, 18].

The totality of the nsSNPs was analyzed by the YASARA software, which allows the visualization of the entire 3D structure of the protein. Among the 8 nsSNPs, 6 were found to be different from the native protein; by the addition or loss of hydrophobic and/or hydrogen bonds. The biochemical differences of the amino acid variants such as hydrophobicity and charge can affect the protein structure due to deleterious nsSNPs [44, 49, 50]. In addition, hydrogen and salt bonds' disruption can alter normal protein function [49, 51].

Indeed, each amino acid has its own charge and specific hydrophobicity value. Regarding the variants R105W and D100N, the wild-type residues were charged and the mutant residues were neutral. The loss of the wild-type protein charge may lead to the disappearance of interactions with other residues. As for the two variants S46L and R105W, the wild-type amino acids serine and arginine differed in their level of hydrophobicity comparing to the mutant amino acids leucine and tryptophan, respectively; therefore, these variants introduce more hydrophobic residues at these positions.

Among the 6 nsSNPs that were found deleterious and revealed a change in the protein structure, two variants were cited in the literature, the N103K variant was reported in two studies. The first one concerned an Egyptian two-year-old homozygous patient with a very low serum Leptin level and a severe early onset obesity, in which the genetic analysis was performed by the sequencing [43]. The second study was designed as a case control and confirmed the association of the N103K mutation with early obesity in a 10-year-old Pakistani patient who also had a very low serum Leptin level, suggestive of a functional impact of the mutation [52]. The second variant, which is R105W, was reported in a study conducted on an obese patient who presented a reduced serum Leptin concentration and related to morbid obesity [53].

Many polymorphisms of the LEP gene were reported in the literature and results are controversial. Two studies on Tunisian populations found a link between the common LEP −2548G/A genotype and obesity [41, 54]. In addition to this SNP, the A19G variant of LEP gene was associated with higher serum Leptin levels in 30 healthy Caucasian infants younger than 6 months but not in an Italian population including 205 obese patients [55].

From 1997 until today, 8 mutations were revealed in LEP gene all causing extreme obesity in infants, 7 of which interfered with the expression or secretion of Leptin resulting in low to undetectable serum levels of this protein [56]. The eighth mutation was reported in 2015, in a Turkish child. Standard immunoreactive techniques showed elevated levels of the Leptin protein that could not turn on the Leptin receptor, thus confirming a functional Leptin deficiency in this patient Mutations in LEP gene are considered as a rare genetic disorder and result in Leptin deficiency or dysfunction, which generates different obesity phenotypes [18].

In the current study, we were able to predict high impact amino acid substitutions on the Leptin structure using computational tools. From 93 nsSNPs, the variants S46L, G59S, D100N, L103K, R105W, and L161R, were predicted to be deleterious, conserved, decrease stability and alter the protein structure. We believe that further functional studies are needed to directly determine the full effect of these mutant proteins in obesity occurrence.

## 5. Conclusion

*In silico* analysis is currently at the center of the coding and regulatory variants screening concerns related to diseases at the molecular level. In this study, *In silico* analysis targeting functional nsSNPs in the LEP gene was performed to investigate the possible effect of nsSNPs on the structure and function of the Leptin protein. The 6 mutations (S46L, G59S, D100N, L103K, R105W, and L161R) were identified as possible causes of the structural modifications of the Leptin protein, and could probably affect its activity. Indeed, a single mutation (C117S) was found in a functional region of the protein and 2 mutations (R105W, N103K) were previously found in patients suffering from obesity.

## Figures and Tables

**Table 1:** NsSNPs predicted as deleterious by SIFT and POLYPHEN.

SNP	MAF	AA Position	SIFT	Score	POLYPHEN	Score
rs866158426	NA	S46L	Deleterious	0.01	Probably_damaging	0.96
rs200575914	0.00000 (1/245916. GnomAD)	G59S	Deleterious	0	Probably_damaging	1
rs886061972	NA	D61N	Deleterious	0	Probably_damaging	0.96
rs724159998	0.00001 (1/121404. ExAC)	D100N	Deleterious	0	Probably_damaging	0.99
rs28954113	0.005 (27/5008. 1000G)	N103K	Deleterious	0	Probably_damaging	0.99
rs1051206328	NA	C117S	Deleterious	0	Probably_damaging	0.99
rs771956117	0.00001 (1/115652. ExAC)	L161R	Deleterious	0	Probably_damaging	1
rs1332916395	0.00001 (3/246190. GnomAD)	D76V	Deleterious	0	Probably_damaging	0.99
rs199838573	0.00000 (1/246242. GnomAD)	S88C	Deleterious	0	Probably_damaging	0.68
rs1206379074	0.00001 (1/125568. TOPMED)	P90R	Deleterious	0.02	Probably_damaging	0.60
rs1226851396	0.00001 (1/125568. TOPMED)	I95N	Deleterious	0.01	Probably_damaging	0.65
rs104894023	NA	R105W	Deleterious	0	Probably_damaging	0.99

GnomAD: The genome aggregation database. ExAC: The exome aggregation consortium. 1000G: 1000 genomes. TOPMED: The trans-omics for precision medicine. NA: Not available.

**Table 2:** Confirmation of the deleterious nsSNPs by other prediction software.

AA positions	PROVEAN_pred	PredictSNP	MAPP	PhD_SNP	SNAP	PANTHER
S46L	D	D	D	N	D	D
G59S	D	D	D	N	D	D
D61N	D	D	D	N	D	D
D100N	D	D	D	N	D	D
N103K	D	D	D	D	D	N
C117S	D	D	D	D	D	D
L161R	D	D	D	D	D	D
D76V	D	D	D	D	D	D
S88C	D	D	D	D	D	D
90R	D	D	D	D	D	D
I95N	D	D	D	D	D	D
R105W	D	D	D	D	D	D

D: Deleterious, N: Nondeleterious.

**Table 3:** Prediction of change in protein stability using I-MUTANT 2 and Mupro.

Uploaded variation	AA position	Imutant-2		MUPRO					
		DDG		DDG		SVM		NN	
rs866158426	S46L	−0.49	Decrease	−0.417	Decrease	−0.476	Decrease	−0.982	Decrease
rs200575914	G59S	−1.15	Decrease	−0.911	Decrease	−0.782	Decrease	−0.693	Decrease
rs886061972	D61N	−1.06	Decrease	−0.813	Decrease	−0.259	Decrease	−0.761	Decrease
rs724159998	D100N	−2.68	Decrease	−1.09	Decrease	−0.781	Decrease	−0.725	Decrease
rs28954113	N103K	−0.7	Decrease	−1.282	Decrease	−0.73	Decrease	−0.663	Decrease
rs1051206328	C117S	−0.98	Decrease	−1.852	Decrease	−1	Decrease	−0.912	Decrease
rs771956117	L161R	−0.29	Decrease	−2.214	Decrease	−1	Decrease	−0.964	Decrease
rs104894023	R105W	−1.02	Decrease	−1.22	Decrease	−1	Decrease	0.90	Decrease

## Data Availability

All data used to support the findings of this study are included within the article.

## Supplementary Materials

Supplementary Materials Supplementary Figure S1: comparison of the native leptin protein structure and it mutant form G59S. Supplementary Figure S2: comparison of the native leptin protein structure and it mutant form N103K. Supplementary Figure S3: comparison of the native leptin protein structure and it mutant form R105W. Supplementary Figure S4: comparison of the native leptin protein structure and it mutant form L161R.
