# Supplementary material for: Prediction and Structural Comparison of Deleterious Coding Nonsynonymous Single Nucleotide Polymorphisms (nsSNPs) in Human LEP Gene Associated with Obesity
Source: Biomed Res Int. 2019 Dec 4;2019:1832084. doi: 10.1155/2019/1832084 (PMC6913293; doi:10.1155/2019/1832084)
Supplement: Supplementary Materials — Supplementary Figure S1: comparison of the native leptin protein structure and it mutant form G59S. Supplementary Figure S2: comparison of the native leptin protein structure and it mutant form N103K. Supplementary Figure S3: comparison of the native leptin protein structure and it mutant form R105W. Supplementary Figure S4: comparison of the native leptin protein structure and it mutant form L161R. [file 1832084.f1.pdf]

Supplementary figures

Color legend : Residues substituted are showed in red, residues involved in hydrogen bonds are marked in magenta, residues participate in hydrophobic interactions are indicated in blue, the residues which lost a hydrogen bonds and/or hydrophobic interactions are marked in green, the new residues appeared are indicated in cyan. Hydrogen bonding are marked by yellow dashed lines and hydrophobic interactions are showed by grey and green lines.

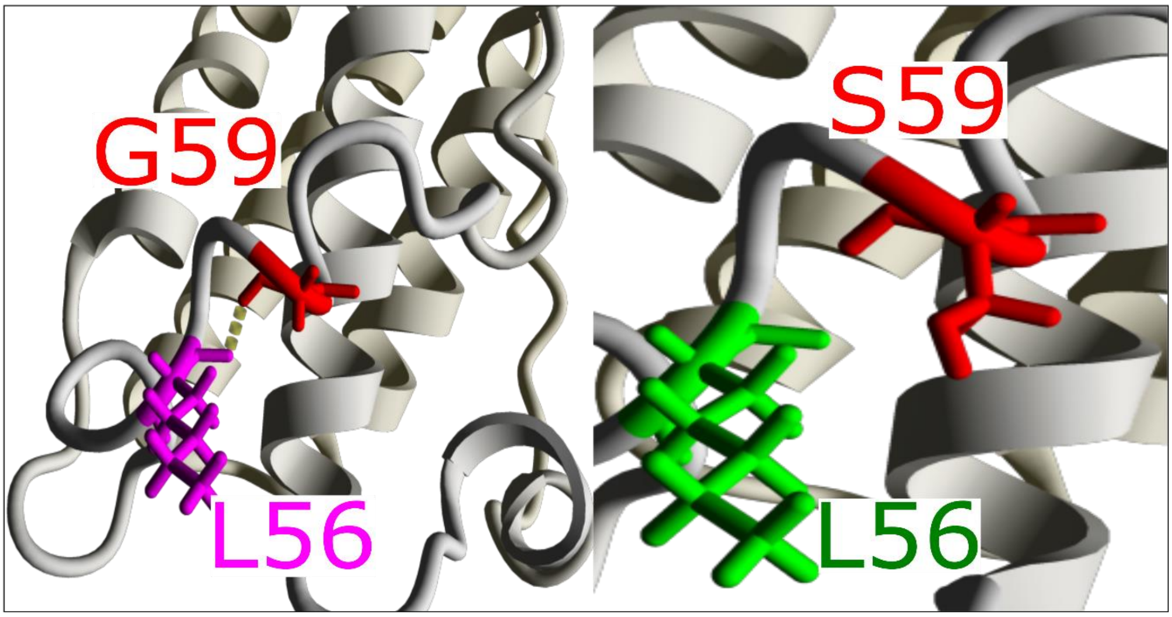

Figure S1 : Comparison of the native leptin protein structure and it mutant form G59S.

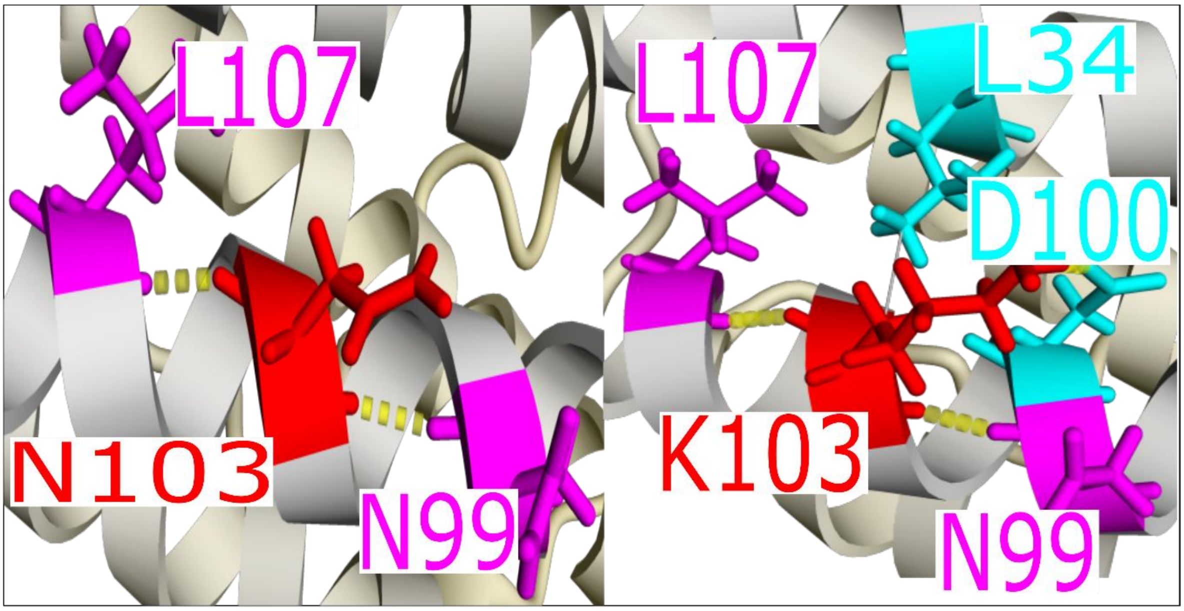

Figure S2 : Comparison of the native leptin protein structure and it mutant form N103K.

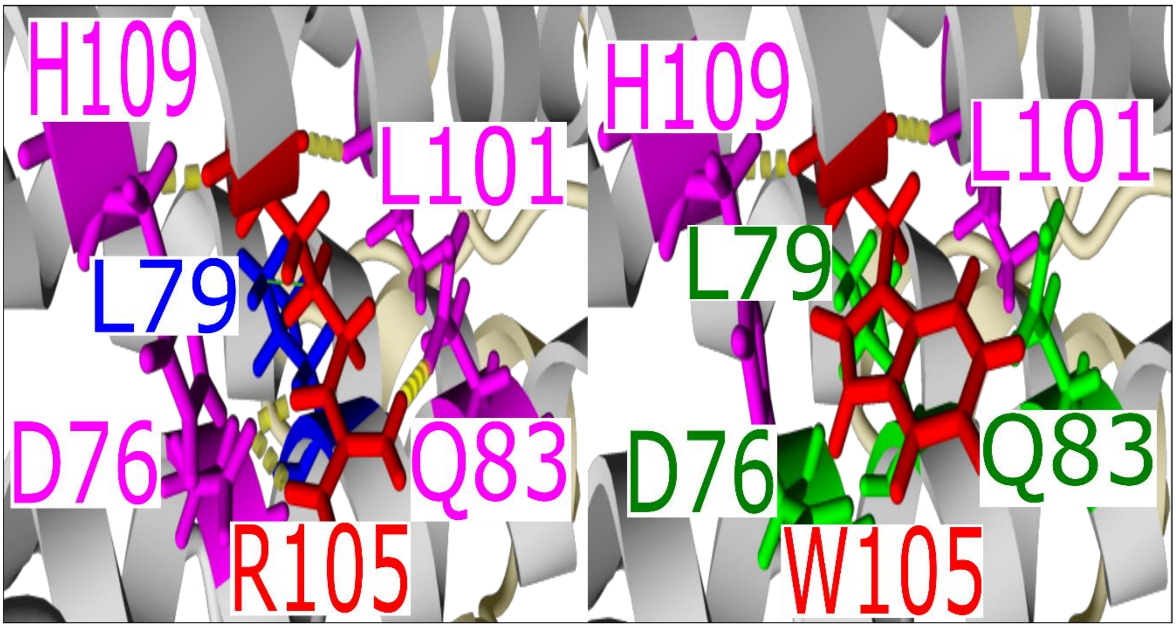

Figure S3 : Comparison of the native leptin protein structure and it mutant form R105W.

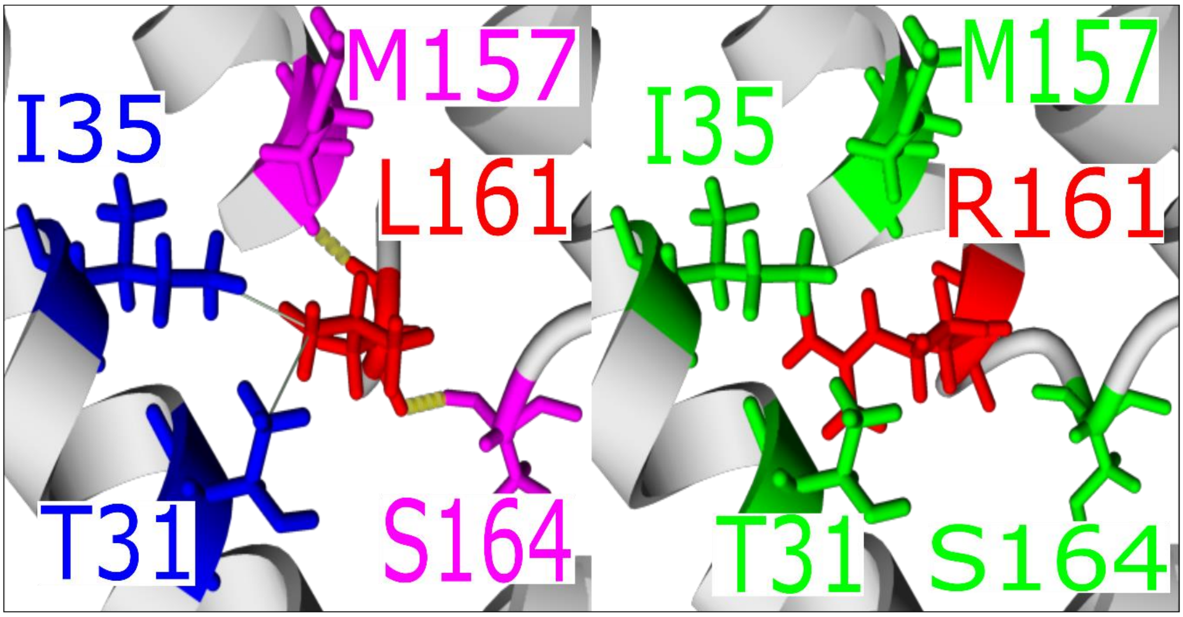

Figure S4 : Comparison of the native leptin protein structure and it mutant form L161R.
